# Effects of Attitudes towards Exercise Behaviour, Use of Sports Apps and COVID-19 on Intentions to Exercise

**DOI:** 10.3390/jpm12091434

**Published:** 2022-08-31

**Authors:** Peng Gu, Zeheng Liang, Hao Zhang, Dazhi Zhang

**Affiliations:** 1 School of Media and Communication, Soochow University, Suzhou 215031, China; 2 School of Physical Education and Sports, Soochow University, Suzhou 215031, China

**Keywords:** sports apps, intention to exercise, COVID-19, attitude, TPB

## Abstract

The sudden outbreak of the novel coronavirus pandemic in 2019 disrupted the normal order of life and work, and the virus is still a major threat prevailing the globe. Confronted with the unknown virus, citizens have been following government policies of COVID-19 treatment and containment, and actively improving their immunity through physical activity (PA). This paper is concerned with ways to guide or promote people’s willingness to exercise, one of the most effective means to boost immunity. Based on the “attitude–intention” correlation defined in the theory of planned behaviour (TPB), this study, by synchronizing online data about workouts, explores the influence of people’s attitudes towards PA behaviour in promoting their intentions to engage in such behaviours as a means to fight the pandemic. In addition, the attitudes towards the use of sports apps and the epidemic are also reckoned with to investigate influencing factors promoting physical activity during the lockdown. The results of the study have been derived from the data of 1223 valid questionnaires, which are subjected to hierarchical regression analysis. Attitudes towards exercise and the use of sports apps are proven to have a significant impact on PA intentions, and the two variables are in direct proportion, with more positive attitudes leading to higher intentions; in contrast, attitudes towards the epidemic do not exhibit an obvious effect. In this light, it is advisable that when clinicians treat COVID-19 patients and medical departments respond to the epidemic, they actively make affirmative influences on peoples’ attitudes towards exercise and formulate appropriate exercise plans based on indicators detected and recorded by sports apps such as vital capacity, heart rate, respiratory index and self-perceived intensity to help them face the risk of the epidemic with more confidence.

## 1. Introduction

In November 2019, in the face of the outbreak of COVID-19, a disease able to be spread among humans, international organizations and countries across the globe proposed or imposed containment measures such as social distancing, lockdown and home confinement. Consequently, sports and workout plans terminated with the closure of gymnasiums and outdoor sports venues, resulting in more sedentary time and less physical activity (PA) [1,2].

In light of this situation, researchers have started to work on health-related behavioural changes, revealing that negative signs in mental health, such as anxiety, depression, tension, anger and fatigue, are associated with lower PA levels. When comparing their pre- and post-pandemic life, 47.4% of those surveyed reported a remarkable reduction in physical activity, and the decrease coincides with higher scores in tests related to negative emotional changes [3]. Despite the “scarcity” of social distancing in the present-day Internet era, when people are explicitly informed of the need to keep their distance, a sense of resistance is generated, and further, lower levels of tolerance to this policy are related to less physical activity [4], including a decrease in sports time. Studies have also shown an association of social distancing with both the curtailed amount and lowered intensity of practising PA [5]. Prior to the outbreak, the World Health Organization (WHO) pointed out that about one in four adults—and the same proportion of adolescents—worldwide failed to meet the global recommended levels of physical activity proposed by the organization [6]. To make things worse, economic growth around the world has, by virtue of technological advances, brought about not only convenience but also excuses for not exercising.

To put it simply, it had been a matter worthy of concern how to improve people’s PA levels before the confinement due to the pandemic. Then, the emergence of COVID-19 undoubtedly exacerbated the unsatisfactory level of sports participation. On this ground, this study sets out to investigate how to promote or maintain the level of physical activity and exercise during lockdown periods. Previous studies have shed light on the various negative effects of a lack of PA and a sedentary lifestyle on human fitness, covering muscle, cardiovascular functions, metabolism and endocrine levels, as well as mental health [7]. In this light, maintaining adequate physical activity and exercise is a cost-effective approach to life in a risky society.

A primary concern of this study is the influence of people’s attitudes towards PA behaviour on promoting their willingness to engage in such behaviours to fight the epidemic. In addition, with the limitation of practising PA due to lockdown restrictions taken into account, which forces people to find another way to exercise, the attitudes towards the use of sports apps and the epidemic are also considered in the study to ensure the validity of the results. To this end, the “attitude–intention” model from the theory of planned behaviour (TPB) is applied, mainly to bring out the relationship between the attitudes toward using sports apps and the intention to fight the epidemic by way of PA behaviours.

## 2. Literature Review

According to the World Health Organization (WHO) classification of physical activities, they fall into three brackets as light, moderate and vigorous in intensity. The WHO also defined the standards of sports needed for different age groups. For example, it is recommended that adults conduct at least 150–300 min of moderate-intensity aerobic activity per week, or at least 75–150 min of high-intensity aerobic activity, or a combination of moderate- and vigorous-intensity activities equivalent to the above amount. Children and adolescents aged between 5 and 17 are recommended to keep an average daily amount of moderate-intensity activity for 60 min [8]. Since the coronavirus outbreak, the WHO has been emphasizing the importance of physical activity, suggesting that regular exercise can prevent health-related diseases [9]. Under the limited conditions, while the benefits of not remaining seated all the time on physical health are obvious, research has also identified positive effects of walking on human health, both physical and mental. Participants of a study who walked 10,000 steps or more per day had significantly lower scores on subscales of anxiety, depression, anger, fatigue and confusion, as well as in the general level of depression [10].

With a lack of time alleged as a major barrier to engaging in physical activity, it should also be noted that a weekly amount of PA of a mere 30 min has proven to be effective in improving health, which may help inactive people adopt a more active lifestyle [11]. The epidemic lockdown has led to increased inertia among people who have become more unwilling to perform PA and have prolonged sedentary time. Therefore, it is more applicable to the current situation to promote behavioural changes by altering people’s attitudes towards sports and exercise than to force the change at the physical level. The theory of planned behaviour (TPB) has evolved to be one of the most commonly used theories for predicting PA behaviours [12], which can help derive more convincing research results in the discussion of how to motivate people to practice PA. Accordingly, this study adopts the behavioural attitude and behavioural intention as two main concepts herein.

### 2.1. Behavioural Attitude and Behavioural Intention

The theory of planned behaviour (TPB) started as one based on a “attitude–behaviour” relation and has gradually developed into a research model of behavioural attitudes, subjective norms and perceived behavioural control. Specifically, the main rationale of the theory is that behavioural attitudes, subjective norms and perceived behavioural control take shape under the guidance of behavioural beliefs, which leads to the generation of behavioural intentions [13]. As an abstract manifestation of a person’s subjective will, attitudes are often included in projects that study certain decision-making or action-taking processes. It is a stable and lasting psychological concept that can effectively influence and predict human behaviour [14].

The TPB is based on a hypothesis that behaviour is predictable by way of intention, and that when a behaviour is in line with one’s own values, the person is inclined to act on the related intention. Attitude is the most straightforward characterization factor of values, which represents the tendency of a person to take on a certain degree of approval or opposition to something or to the performance of an act. In short, a person who believes that performing an act will mostly produce positive results has a positive attitude towards this act, and vice versa. According to a review of the application of the TPB to health-related behaviour, behavioural intention is an important predictor of behaviour and attitude of intention [15]. In this light, behavioural intention and behavioural attitude proposed in the TPB constitute a major logic for relevant research.

The theory has been used by many scholars in the fields of consumer choice, environmental protection, health promotion, tourism development, among others. For studies on sports, it is often suggested to use devices that can visualize data from the behaviour, such as accelerometers and trackers, when PA or exercise behaviours are concerned, because this can help to draw conclusions closer to reality, more objective and more scientific. Yet, it appears uneasy to actually put such devices into use during the current “screen-to-screen” era and a time of social distancing due to the epidemic. In a study using the TPB to explore the promotion of autonomy for participating in PA among primary school students, intervention in attitudes is proven to be conducive to student participation [16]. On this basis, this study is mainly concerned with the impact of behavioural attitudes on behavioural intentions as postulated in the TBP.

Attitude refers to an individual’s positive or negative evaluation of a particular phenomenon or behaviour [17]. A study on the influence of risk knowledge on rural tourism intention during epidemic containment shows that a risk-averse attitude has a notable impact on people’s intention with regard to rural tourism behaviour [18]. Another study on travel intentions conducted in Hong Kong also manifests that when risk attitudes decrease, travel intentions increase [14]. The attitude, a factor with a certain degree of stability, can thus serve to predict individual behavioural intentions [19]. Therefore, the exploration into people’s attitudes towards PA behaviours, sports apps and the epidemic can shed light on people’s intention to perform such behaviours to resist the epidemic.

### 2.2. Physical Activity (Exercise), Sports Apps and COVID-19

Under the rationale of “attitude–intention” relation in the TPB, the study is carried out in two separate phases and the results are subject to comparative analysis. The first-phase study is focused on the same behaviour, investigating people’s attitudes towards PA behaviours and their intentions to practice such behaviours to fight the epidemic; the second phase deals with different behaviours, probing into attitudes towards using sports apps and the COVID-19 pandemic.

A study spanning 14 countries reveals a significant drop in PA levels globally during the COVID-19 pandemic, and the self-report data shows that 41% of the 13,503 respondents have experienced reduced moderate- and vigorous-intensity physical activity [20]. Similar results can also be found in research from countries such as Italy and Spain, where participants, when surveyed about PA levels during confinement periods, self-reported a significant increase in sedentary time and a decrease in physical activity [2,21]. Meanwhile, time spent on electronic devices rose while that spent walking was shortened [2]. Research by Cristina et al. demonstrated the same conclusion—sedentary time has seen a rise [22].

According to the WHO’s World Health Statistics 2021 Report, seven of the top ten causes of death in 2019 were chronic non-communicable diseases, and the corresponding death rate increased from 60.8% in 2000 to 73.6% in 2019 [23]. It elevates the need for studies on the promotion of physical activity, considering the importance of PA as a feasible personal health behaviour that can effectively improve the quality of life and prevent diseases. All forms of regular PA of sufficient duration and intensity can provide health benefits [6], and studies have also shown that regular PA behaviours are one of the most effective ways to prevent premature death [24].

After the prevalence of the H1N1 influenza, there is growing epidemiological evidence that physical activity prior to infection has a dose-response relationship with the incidence, duration or severity of self-reported and laboratory- or hospital-diagnosed acute upper respiratory infections [25]. In short, the performance or intensity of PA has an impact on respiratory infections such as COVID-19. Appropriate PA behaviours can enhance the monitoring function of the human immune system. Chagas et al. have also pointed out that systematic exercise has a positive effect on strengthening the immune system and reducing the risk of diseases [26].

Epidemic containment policies—home confinement, for instance—have also exerted certain effects on people’s PA behaviours, and it is particularly necessary to find alternatives to maintain PA levels under such conditions. Caught off guard by the epidemic, people have taken health as a measure of life satisfaction. Based on this change, and in light of the TPB “attitude–intention” rationale, we have put forward the following questions and assumptions regarding people’s attitudes towards PA behaviours:

RQ1: Do people’s attitudes towards PA behaviours correlate with their intentions to engage in such behaviours?

**Hypotheses** **1** **(H1).** *More positive attitudes toward PA behaviours coincide with higher intentions to practice PA behaviours.*

Based on the current situation of terminal applications of mobile Internet technologies and smartphones, sports apps were a concern of this study as an auxiliary tool for workouts. During the confinement when people work and study from home, the Internet can be a useful resource for transmitting information to promote health, and international organizations and institutions around the world have indeed put this resource in use [2]. For example, the WHO released guidelines for “Stay Physically Active during Self-quarantine”, the Italian Ministry of Health issued “COVID-19, How to Follow an Appropriate and Healthy Lifestyle When Staying at Home” to guide Italian people to exercise at home, and the Information Center of the General Administration of Sport of China launched an information retrieval platform for at-home workout methods during epidemic containment and control, which has gathered and screened more than 1500 at-home fitness methods and relevant knowledge, aiming to publicize proper at-home workout techniques and enrich people’s life during home quarantine [27,28,29].

After China began to implement home isolation measures, the Institute of Sport Science of the General Administration of Sport of China and sports APP Keep jointly launched the “At-home Fitness Program for COVID Containment” [30]. The program incorporates a number of items, including cardiopulmonary function activation training, Tai Chi fit and 18 scientific fitness methods, which are intended to help people keep exercising beyond the barriers caused by the confinement. The APKPure APP also provided a guide for at-home workouts to win the campaign against COVID-19. Studies have shown that the use of smartphone apps and trackers can significantly predict PA levels as classified as light, moderate and vigorous, and respondents also reported that they were more willing to use smartphone apps in the future [31]. Similar results have been seen in studies on recently retired adults, which reveal increased PA levels brought up by e-health interventions [32].

The “2020 Home Fitness Short Report of China” released by Leadleo.com (accessed on 12 March 2022) shows that from 25 January to 25 February 2020, the daily downloads of Keep, Daily Yoga and Mint Health apps all achieved substantial growth, while Keep had the highest increase, reaching 478% [33]. In addition, from 20 January to 2 March 2020, the search index [Baidu Search Index (BSI): Internet users’ interest in keyword search and continuous changes. Algorithm Description: Based on the number of searches on Baidu among netizens, the keywords were analyzed as statistical objects and the Baidu search frequency weight for each keyword was calculated.] for the keyword “Keep” rose from 3444 to 11,623, and the search volume of other keywords such as yoga and aerobics also tended upward during the same period. Judged from existing facts, people’s attitudes towards sports apps during the lockdown periods are relatively positive.

In another study aimed at increasing PA levels in breast cancer survivors, based on data proving that PA has positive effects on breast cancer treatment, including mitigating symptoms and improving the quality of life for survivors, researchers relied on the mobile APP MapMyFitness to track and record PA behaviours and set PA programs, and social media Facebook (now Meta) to send twice-weekly health reminders. The study enabled breast cancer survivors to participate more actively in physical activity, resulting in improved mental health and quality of life [34]. All in all, from e-health interventions in the past to the use of apps and social media during the current days, such approaches have been useful in removing the various barriers people encounter in the process of practising PA and social distancing, which have been posed as barriers, is also lifted by the use of sports apps, which is expected to promote individual PA behaviours.

Furthermore, a review on the role of smartphones in promoting PA points out that smartphone-based interventions can effectively raise the number of steps per day in adults, thereby improving PA levels, while APP-based interventions have the same influence, increasing both time and daily steps [35]. The use of the APP B-MOBILE has been proven to markedly increase the time spent on light and moderate-to-vigorous physical activity [36], and a week’s use of stAPP is also proven to be effective in resulting in more times people rest after a long time of sitting, thereby reducing sedentary time [37], and the number of fans on relevant apps, the quantity of other materials users follow, and the times that users are motivated by others all contribute to the attainment of the goal [38].

China has been encouraging enterprises to participate in the construction of national fitness technology innovation platforms and fitness guidance platforms [39] and the sports APP Keep was announced to be listed in Hong Kong on March 15, making it the “first domestic online fitness stock”. It can be seen that online fitness has become a new development strategy and trend under the current circumstances, whether in terms of national policies, market ecology or corporate innovation. At the same time, many studies have proven the positive impacts of sports apps on PA, and it is natural for those apps to be popular as technical carriers of interventions in PA levels during special times. In this light, the study has taken the “attitude–intention” rationale in the TPB as a basis and proposed questions and hypotheses around people’s attitudes towards using sports apps:

RQ2: Do people’s attitudes towards using sports apps correlate with their intentions to engage in such behaviours? How can we adjust the relationship between PA attitudes and PA intentions accordingly?

**Hypotheses** **2** **(H2).** *More positive attitudes towards using sports apps coincide with higher intentions to practice PA behaviours.*

As of 23 January 2022, more than 346 million confirmed cases and more than 5.5 million deaths had been reported globally. On 30 January 2020, the WHO declared the outbreak a “public health emergency of international concern” (PHEIC). The negative impact and, even worse, the threat to life that COVID-19 has brought across the globe makes it reasonable for individuals to consider it a risk. Past experience has taught us the lesson that the successful control of the spread and development of infectious diseases depends to a certain extent on the public’s sound understanding of individual and social risk factors [40]. Simply put, people’s attitudes towards the outbreak can influence their behaviours and actions in response to it. In our study, people’s attitudes are assumed to be more positive when they perceive the risk of the epidemic more actively, and exercise is deemed as an individually executable health behaviour that improves the quality of life and prevents the disease. Under the rationale of the “attitude–intention” relation in the TPB, we have also brought forth questions and hypotheses as regards people’s attitudes towards COVID.

RQ3: Do people’s attitudes towards the epidemic correlate with their intentions to engage in PA behaviours? How can we adjust the relationship between PA attitudes and PA intentions accordingly?

**Hypotheses** **3** **(H3).** *More positive attitudes towards the epidemic coincide with higher intentions to practice PA behaviours.*

## 3. Materials and Methods

### 3.1. Research Design

Based on the “attitude–intention” link in people’s behaviour designated in the theory of planned behaviour (TPB), this paper explores the factors that affect exercise intention in risky environments from the two dimensions of behavioural restriction and psychological interference. Specifically, the intention to resist the epidemic by conducting exercise behaviour was taken as a dependent variable, and the independent variables included the attitude towards exercise behaviour, the attitude towards the use of sports apps and the attitude towards the epidemic. This study was mainly guided by the method of multiple regression analysis and conducted in two phases.

The first stage aimed to determine whether the impacts of the attitude towards the same behaviour and of the attitudes towards other behaviours on the certain behavioural intention are consistent. This phase firstly dealt with the “attitude–intention” correlation when the same behaviour was concerned. Accordingly, the attitude towards exercise behaviour was the independent variable and its influence on the dependent variable of the intention to adopt the corresponding behaviour in response to the epidemic. Secondly, with regard to the “attitude–intention” correlation in terms of different behaviours, the attitude towards the use of sports apps and the attitude towards the epidemic were adopted as independent variables to investigate their impact on the dependent variable of the intention to take exercise behaviour to resist the epidemic.

**Figure 1:**
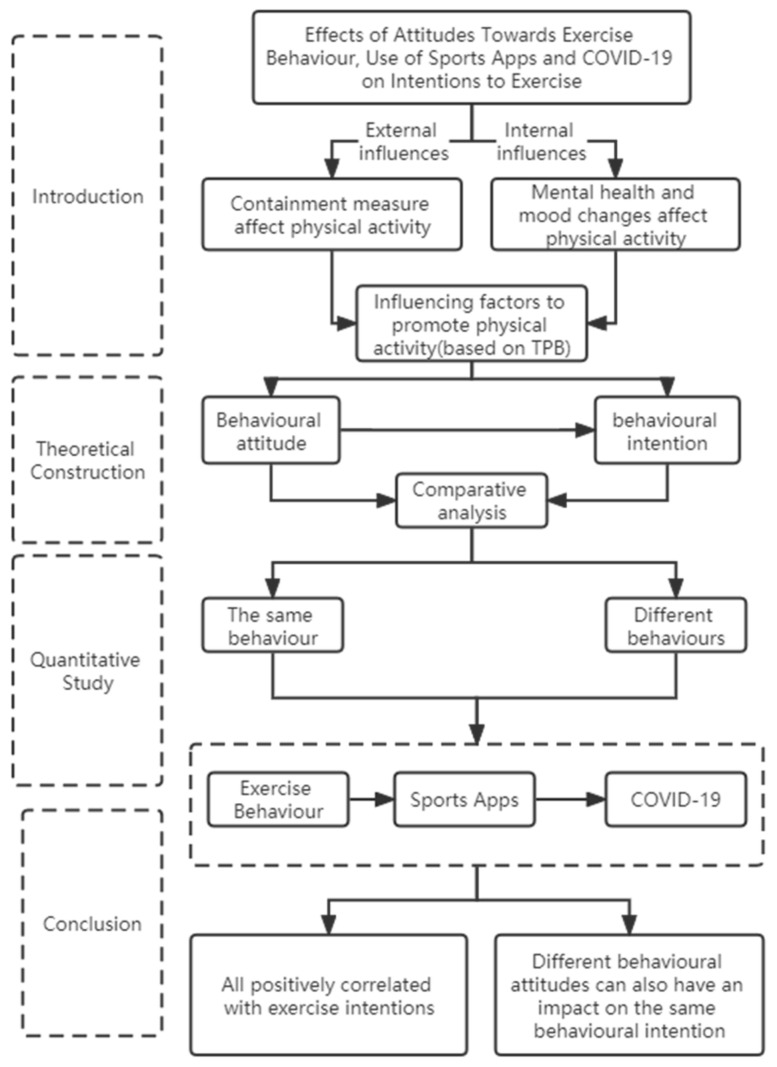
Flow chart of the research design.

During the second stage, with respect to the same behavioural intention, the study analyzed whether attitudes towards different behaviours have moderating effects on the “attitude–intention” link in certain behaviour. The analysis was conducted by virtue of hierarchical multiple regression in three steps. The first step adopted demographic variables as independent variables to examine their influence on the intention to resort to exercise behaviour as a means against the pandemic; the second step introduced an additional independent variable of attitude towards exercise behaviour and further analyzed the impact on the dependent variable; in the third step, another two independent variables―attitude towards the use of sports apps and that towards the pandemic―were added on the basis of the previous two steps to determine whether attitudes towards different behaviours have moderating effects on the “attitude–intention” correlation in certain behaviour. The specific flow chart is listed in Figure 1.

### 3.2. Sample and Procedure

Following the requirements of national epidemic containment policies, this study adopted the approach of a non-contact questionnaire survey for sample collection, conducted in mid-January 2022, under the assistance of a survey company. As of 16 January 2022, 1600 participants completed the questionnaire. The survey was anonymous, ensuring the privacy of the participants. No human experiment or identifiable human data collection was involved. Invalid questionnaires were screened out based on “whether the participant has used sports apps”, and the total number of valid samples obtained was 1223. Among these, 45.4% were male participants and 54.6% female, the proportion of which is in line with the gender ratio in the Yangtze River Delta region identified in the “2021 China Statistical Yearbook” compiled by the National Bureau of Statistics of China. The vast majority of the participants came from the Yangtze River Delta region (including Jiangsu, Zhejiang and Shanghai). Considering that the economic development of this area is relatively ahead of other parts of China, and people’s living standards are higher as well, the pursuit of a digital and smart life is more evident. According to the “White Paper on China’s Sports and Fitness Industry Development Trends in 2019” released by iResearch, users of sports and fitness apps in first- and second-tier cities accounted for a major proportion, and the average age was younger, so the sample collected in the Yangtze River Delta region could produce more convincing results.

It should be noted that participants have shown special attention to PA levels after the outbreak of COVID-19: 81.8% of the participants believe that during home confinement, it is better to be physically active than to remain sedentary; 69.4% of them have paid increased attention to fitness-related indices; and 16.8% confessed that they used to not exercise before COVID-19, and started exercising afterwards. According to the standard of PA levels recommended by the WHO (at least 150 min of moderate-intensity exercise or 75 min of vigorous-intensity exercise per week), only 50.4% of the participants in the adult group met the standard prior to the outbreak, and this figure rose to 61.4% subsequently. Demographic variable-related data are listed in Table 1.

### 3.3. Measurement

In addition to demographic data, we also surveyed participants’ attitudes towards PA behaviours, use of relevant apps and the pandemic, as well as their intentions to perform PA behaviours to resist the pandemic. Participants were asked to fill out a five-point Likert scale form (1 = ‘strongly disagree’, 5 = ‘strongly agree’) by selecting the corresponding item for each question that matched their personal reality. Before the survey, we invited experts from relevant professions to propose suggestions on amending the questionnaire, which was modified accordingly before we proceeded to give out the questionnaires. Descriptive statistics and correlation coefficients for key variables are presented in Table 2.

**Intention to resist the pandemic by practising PA behaviours.** In Italy, the first European country to enter a national lockdown, researchers investigated the psychological status (including depression and anxiety) and sleep disorders of Italians during the confinement and found that the rates of depression and anxiety were 24.7% and 23.2%, respectively, while 42.2% of the people had sleep disorders [41]. A study on healthcare workers in Spain revealed that more than one-fourth of those personnel experienced anxiety, depression and emotional depletion during the epidemic [42]. In the above discussion, it has been shown that regular physical exercise is beneficial to people’s physical and mental health. Therefore, two items related to exercise intentions during the pandemic are set in the questionnaire: (1) During the pandemic, I want to maintain a healthy body through exercise; (2) During the pandemic, I want to relieve stress and anxiety by exercising so as to improve sleep (Cronbach’s alpha = 0.974).

**Attitudes towards PA behaviours.** “White Paper on China’s Sports and Fitness Industry Development Trends” summarizes the internal and external factors influencing China’s sports and fitness population, including the pursuit of health and physical fitness and the relief of stress, and the requirements of body shape. Accordingly, the questionnaire also includes the following four items set in the context of COVID-19: (1) I think exercising will help me get a good figure; (2) I think exercising will enrich my social life; (3) I think keeping exercising will improve my immunity; (4) I think keeping exercising will reduce my chances of contracting COVID-19 (Cronbach’s alpha = 0.905).

**Attitudes towards using sports apps.** The “Research Report on China’s Intelligent Sports and Fitness Industry in 2021” released by iResearch divides sports and fitness apps into three categories: sports and fitness service apps (e.g., Keep and APKPure), intelligent mobile terminal auxiliary apps (e.g., Mi Sports and Apple Health) and sports brand apps (e.g., Nike Training Club and Adidas Go). Through real user experience of functions and services provided by top sports apps on the market, the basic characteristics of sports apps with distinct features and commonalities are sorted out. Based on this, three items were set when questioning the participants’ attitudes towards using sports apps: (1) I want to use sports apps to access diversified fitness courses/health knowledge; (2) I want to use sports apps to record exercise data and analyze monitoring data; (3) I want to use sports apps to customize diet plans and training plans (Cronbach’s alpha = 0.936).

**Attitudes towards the pandemic.** In this study, people’s attitudes are assumed to be more positive when they perceive the risk of the epidemic more actively. Risk perception refers to people’s intuitive assessment of their exposure or potential exposure to danger [43]. COVID-19 was forced into people’s lives as an unknown virus. In the early stage of the outbreak, there was neither a clear detection method nor a targeted drug and the unpredictable development of the situation aroused people’s fear of the newly emerged matter. This is a subconscious sense everyone may have, and it can drive us to act cautiously or actively to take measures to help defuse fears or risks. Furthermore, the virus spreads invisibly but alerts people with a visible mortality rate. In this regard, three questions are proposed to explore people’s attitudes towards the pandemic: (1) I think the impact of the epidemic is beyond my cognitive expectations; (2) I have a fear of the epidemic; (3) I think the epidemic is deadly (Cronbach’s alpha = 0.729).

Validity analysis can be used to test the accuracy of construct measurement, i.e., the validity of the scale. The Kaiser–Meyer–Olkin (KMO) value in this study was 0.908, and the results of the Bartlett test (X^2^ = 14,385.36, df = 66, *p* < 0.001) were significant. The KMO value for the PA intentions variable was 0.50, for attitudes towards PA behaviours 0.754, for attitudes towards using sports apps 0.781 and for attitudes towards the pandemic 0.5. These data demonstrate the validity of the scales in this study.

## 4. Results

### 4.1. Descriptive Results and Correlations

Through a descriptive analysis of variables (Table 2), we learned that the participants had relatively high intentions to adopt PA behaviours to resist the epidemic (M = 3.944; SD = 0.708), and attitudes toward PA behaviours (M = 3.953; SD = 0.654) and attitudes toward using sports apps (M = 3.980; SD = 0.728) were all positive. For the gender (M = 0.455; SD = 0.498) variable, males had higher PA intentions and attitudes toward PA behaviours than females, which is consistent with results of previous studies. The population using sports apps can be considered to be more receptive to new things or new approaches to exercise. Among the participants in this study, middle-aged and older adults accounted for about 25%, and this, to a certain extent, shows that the popularity of digitalized and intelligent life is on the rise. Attitudes toward the pandemic (M = 3.545; SD = 0.853) showed slightly lower positivity. Cities in the Yangtze River Delta region have adopted fairly strict COVID-19 containment measures, thus, to some degree, neutralizing the attitudes towards the epidemic self-reported by participants in the questionnaire survey. It is gratifying that the participants acknowledged the Chinese government’s capacity for epidemic containment and control to a certain degree. On the whole, the independent variables and the dependent variable were statistically correlated. When the independent variables were entered into the regression equation, no correlation was found between the two variables >0.80, and the variance inflation factor (VIF) was not significantly higher than 3.

### 4.2. Multiple Linear Regression

This study divided the hierarchical multiple regression analysis into two phases. The first phase was to confirm the “attitude–intention” rationale in the TPB in terms of attitudes toward the same behaviour (exercise) and different behaviours. The results of this phase of research were consistent with previous studies with regard to the same behaviour, but disparities were seen for different behaviours. The second phase was to conduct a hierarchical regression analysis on three variables, mainly to investigate whether the attitudes toward using apps and the epidemic had a moderating effect on PA attitudes and intentions. Independent variables of attitudes toward different behaviours were taken into account to render a more comprehensive investigation into the impact of attitudes on intentions and also to explore more dimensions for promoting PA behaviours among people.

The phase-1 analysis results (Table 3, Model I) significantly verify the logic of “attitude–intention” in the TPB. First of all, in Step 1, the demographic variables were examined, the results of which demonstrate that the prediction effect of the gender variables was remarkable. At the same time, the intention of men to perform PA behaviours to resist the epidemic was higher than that of women (β = 0.592, *p* < 0.001), and the model of demographic variables generated distinct results: F(31,219) = 221.57, *p* < 0.001, which makes up 35% of the variance in intention, and the change in R2 noteworthy. In Step 2, the PA attitude was added to the previous model, and it significantly increased the variance (F(41,218) = 1505.53, *p* < 0.001), explaining the intentions of the participants to take PA behaviours to resist the epidemic, accounting for 48% of the variance in intention. Therefore, for RQ1, a substantial correlation was present between PA attitudes and intentions, and H1 was also confirmed. Thus, the conclusion is drawn that the more positive attitudes people have toward PA behaviours, the higher their intention to practice such behaviours. The theoretical logic assured in the TPB that attitudes promote the generation of intentions has also been verified, but it is focused on the attitude and intention regarding the same behaviour. In order to enrich this rationale, this study attempts to explain the same behavioural intention with multiple behavioural attitudes. Accordingly, the impact of attitudes towards apps and the epidemic on PA intentions is examined herein.

According to the results of Mode II and Mode III, it is evident that the significance of the influence of demographic variables in the model was consistent. In Mode II, it can be seen that 23% of the variation in the intention to perform PA behaviours was caused by the variable of attitude towards apps (F(41,218) = 425.63, *p* < 0.001), and H2 is thus confirmed; that is, the more positive attitudes people have towards the use of sports apps, the higher their intention to practice PA behaviours. In Mode III, although the attitudes toward epidemic (F(41,218) = 195.04, *p* < 0.001) present positive effects on the willingness to perform PA behaviours, the effectiveness of contribution was low, so the explanatory power of H3 is limited. To sum up, based on and beyond the TPB, different behavioural attitudes can also have significant impacts on the same behavioural intention, but the explanatory power differs among the specific effects.

Since the influence of each variable in the first-phase analysis was significant, in the second phase, all variables were put into the same model for hierarchical multiple regression analysis (Model IV), which exhibits predicted results of performing PA behaviours to resist the epidemic (Table 4).

Based on Model I in the first phase, the second-phase Model IV supplements Step 3 (Table 4), adding attitudes toward apps and the epidemic to the regression model, and the results show that the gender variable among the demographic variables still has a considerable impact on the model (β = 0.149, *p* ≤ 0.000) (F(3,1219) = 221.57, *p* < 0.001), with the explanatory power accounting for 35%. Attitudes toward exercise constitute the variable (β = 0.778, *p* ≤ 0.000) (F(41,218) = 1505.53, *p* < 0.001) of the most obvious explanatory power, significantly increasing the variance explaining intention changes, accounting for 48%. Although the impact of attitudes toward apps and the epidemic in the previous model is notable (F(61,216) = 1016.28, *p* < 0.001), the explanatory power was low, and the power mainly came from the former variable (β = 0.056, *p* ≤ 0.001), while the *T*-test result of the latter variable (β = 0.016, *p* = 0.199) was insignificant. Therefore, for RQ2, people’s attitudes toward using sports apps are related to intentions to exercise and can serve to moderate the relationship between PA attitudes and intentions, but the degree of adjustment is low. For RQ3, it is only confirmed that there is a certain relation between people’s attitudes towards the epidemic and the intention to practice PA behaviours, but no adjustive functions were found as to the relationship between PA attitudes and PA intentions.

## 5. Discussion

Based on the behavioural attitude–intention rationale proposed in the Theory of Planned behaviour (TPB), this study explores the relationship between participants’ attitudes towards physical activity (PA) behaviours using sports apps and the epidemic and their intention to perform PA behaviours to fight the epidemic. The results show that there is a marked connection between attitudes towards PA behaviours and the use of sports apps with the willingness to practice PA behaviours as a means to resist the epidemic. This firstly demonstrates the relationship between behavioural attitudes and behavioural intentions for the same behaviour, as confirmed in the TPB, and further extends the scope to the relationship between different behavioural attitudes and the same behavioural intention. The results of this study are in line with previous research. In a study on the intervention of sedentary behaviours using smartphone apps (MyHealthAvatar-Diabetes APP), the researchers used the TPB to examine the determinants affecting sedentary behaviours, which found that respondents’ attitudes towards sedentary behaviours played a positive role in implementing the intervention and that the overall intention influenced by attitudes also had a positive effect on changing sitting behaviours [44]. Similarly, Arlati et al. found that VR-based apps could render it possible to perform training with other users, hence reducing the risks due to social isolation [45].

Attitudes towards PA behaviours were, to a significant degree, positively correlated with PA intentions, and this finding reinforces the promotion effect of attitudes to intentions in the TPB. When studying the influencing factors of sports behaviours among female college students in Korea, researchers also found that attitude was a powerful one among those elements [46]. With social media being all the rage today, opinion leaders, as well as families and friends on social media, are influencing people’s attitudes toward themselves and society. Community communication functions and the sharing of sports data and sports goals in sports apps, as previous research has shown, also affect people’s attitudes toward exercise to a certain extent.

We can see traces from relevant practical applications that the more positive the attitudes toward using sports apps, the more likely people will practice PA behaviours. The notion of “online marathon” appeared in China during the second half of 2013. With the continuous development of sports apps, the form of “online marathon”(marathon events without runners being present at the scene but held via the recording and statistics of running miles by virtue of Internet big data and other technologies) was first practised on sports APP APKPure in 2015 and witnessed a boom from 2016 to 2017. In recent years, this notion has been transformed and upgraded to “cloud marathon”. Especially in the era of the epidemic, sports events with tens of thousands of participants are subject to the new principle of “social distancing”, so more “cloud marathon” activities have entered people’s lives, for example, the 2020 China Marathon Relay to Fight Epidemic, the 2020 National Online Vertical Marathon and the Charity Run in Fight of COVID-19. The popularity of “cloud marathon” indicates that sports apps are infiltrating people’s pursuit of survival and life from all directions. It is thus safely concluded that the PA level will be improved to a greater extent when people are encouraged to view sports apps positively and acknowledge the convenience and value of using those apps. An overview of smartphones and health-promoting behaviours states that the use of relevant tools helps prevent chronic deformity and obesity and promotes healthy lifestyles and physical activity [47].

One unexpected result of our study is that men are more likely to develop an intention to take PA behaviours to fight the epidemic, a result in line with several previous study outcomes, presenting statistically significant differences between the two genders in the willingness to exercise, with males scoring higher than females [48]. Numerous studies have shown that there are great gender disparities in physical activity. In Europe, the PA level among teenage women in Spain ranks the lowest [49]. It has been noted that in some countries where resources are invested in achieving gender equality, women are found to engage in more physical activity than men [49]. Therefore, how to strengthen women’s physical activity deserves our attention.

In the two variables added to the regression model in Model IV, the variable of attitude toward the epidemic does not significantly affect or promote the relationship in the previous model. The insignificant difference is also understandable. For one thing, because this study is based on the self-report from the participants, it is difficult to revive the emotional memory of the past, and when the participants filled in the questionnaires, the epidemic had been effectively contained, so their attitudes toward it were easy to neutralize. For another, a study shows that the likelihood of refusing to get vaccinated for the coronavirus is 2.44 times higher for those who are averse to other vaccines [50]. Therefore, people’s attitudes toward the epidemic may also be affected by subjective and objective knowledge; for instance, people’s perception of COVID-19 tends to decrease because of their experience with the 2003 SARS pneumonia.

In the era of big data, the need for data visualization is growing clearer, and more people are self-monitoring and feedbacking on their exercise by using wearable devices in everyday exercise activities. Current wearable technologies can generally monitor PA levels by detecting the wearers’ steps, calories consumed and sitting and workout minutes [51]. For the healthy population, they can make timely adjustments to their exercise plans based on the recorded data to avoid damage to the body and prevent infection of diseases. For others, it is helpful to monitor changes in physical health or in lesion characterization indicators, and the relevant feedback helps doctors better personalize treatments to patients, improve health indexes of patients with chronic diseases and increase their motivation for physical activity [52]. For example, fitness watches, when combined with other lifestyle interventions, can increase PA of moderate and vigorous intensity in adults who are overweight or obese [53], and can also improve the PA levels of breast cancer survivors [54].

It is not difficult to find some tech companies such as Xiaomi and Huawei present in the sports APP market. Wearable and traceable technology innovations and the construction of platforms and development of such apps help better integrate apps and external devices. In future research, attention should be paid to the influence of sports apps and wearable devices on PA behaviours. Narici et al. report that muscle mass decline, heart and lung health degradation and increased mortality caused by long sitting time can be monitored via wearable technologies which detect risks, helping to reduce physical inactivity and ensure improved cardiovascular indicators [55]. On this basis, this study has taken into consideration the influence of the use of sports apps and wearable devices on the prediction of intention. The data show that 86% of those who use sports apps are willing to use wearable devices for more personal data records on sports apps regarding various body parameters or sports parameters, which also signals the possibilities of future research.

## 6. Strengths and Limitations

The main theoretical contribution of this study to sports science research is that it reveals the influence of sports products on people’s intention to maintain health under technical guidance. The relationship between behavioural attitudes and behavioural intentions in planned behaviour theory (TPB) demonstrates that people’s attitudes towards something or certain behaviour can produce relevant behavioural intentions. This study shows that we can motivate people to have higher intentions to practice PA behaviours to resist the epidemic by intervening in their attitudes toward PA behaviours, the use of sports apps and the epidemic, thus enriching the knowledge scope of the TPB. However, the influence of the attitude variable regarding the epidemic on the relationship between PA attitudes and PA intentions is not noteworthy.

The study also has significance in providing guidance for future practices. The sudden attack of the COVID-19 pandemic disrupted people’s daily activities; social distancing or home quarantine has become a new norm in life and digitalization has come into full bloom. The online modality is noticeably seen, such as online education, remote office, livestreaming marketing and resumption of work, production and schooling in the cloud, so using sports apps for guiding physical activity or exercise undoubtedly brings a new direction for innovation to the establishment of epidemic containment and control policies. With the ever-changing setup of the world, despite the insignificant influence of the attitudinal variables with regard to the epidemic displayed in this study, people can actually perceive different degrees of social risk. Along with this, people’s pursuit of health and exercise is increasing, and the pursuit of quality is also rising. Our study has provided a direction of thinking in the context of adaption to the new rhythm of life under social distancing and promotion of the frequency and quality of physical exercise among the population.

The limitation of this study is, first of all, that the study is only applicable to China, with the situation probably being different for other countries, and that the sample of the study is limited to the Yangtze River Delta region, with a relatively narrow power of interpretation of the problem at the national level. In addition, there is a certain degree of inability to ensure the equality of the use of digital technologies in this study. According to the 49th Statistical Report on the Development of the Internet in China released by CNNIC, as of December 2021, the number of Internet users in China was 1.032 billion, and the Internet penetration rate reached 73% [56]. The digital divide between urban and rural areas and between generations persists, although the penetration of mobile and Internet technologies in China has been expanding, and the number of Internet users climbing. Furthermore, it has been more than two years since the emergence of COVID-19, so the answers collected about the attitudes, workout time and sitting time during the lockdown from January to March 2020 are backward-looking, which might lead to the problem that the answers from the respondents were likely to be influenced by blank or vague memories when the form was filled out. Finally, it is believed that in order to more properly present relevant influencing factors of physical activity, big data technologies and necessary technical equipment such as trackable and wearable devices should be employed.

## 7. Conclusions

In the global prevalence of the COVID-19 pandemic, people have found that no matter how strong individuals could possibly be, they can fail to withstand the risk of infection, not to mention the elderly and vulnerable people with poor health or immune deficiencies. Therefore, marginal topics such as “physical activity” and “exercise” behaviours, which have formerly been much overlooked, have returned to the research perspective of scholars. Since the outbreak, there have been countless articles published with the keyword of “COVID-19”, which have provided a perspective for national decision making, and meanwhile promoted the decoding of the unknown. This study follows the attitude–intention logic defined in the planned behaviour theory (TPB), and mainly explores the influence of attitudes toward exercise, the use of sports apps and the epidemic on people’s intentions to practice physical activity behaviours to resist the epidemic. It is found that the three attitudes are all positively correlated with exercise intentions to a significant extent, while the power of explanation of the attitude towards the epidemic is relatively low. Overall, the results are consistent with previous studies related to the TPB and serve to enrich the range of the theory; that is, different behavioural attitudes can also have an impact on the same behavioural intention. This, without doubt, presents an effective way to maintain exercise in this particular period. At the same time, the study also throws new light on COVID-19 treatment and containment policy making for clinicians, medical departments and government departments—it is desirable to emphasize the necessity of exercise in the face of epidemic risks, boost people’s positive attitude toward exercise and make appropriate and feasible exercise plans with the help of indicators such as cardiorespiratory endurance, heart rate and pulse and respiratory index recorded in sports apps, which are under constant improvement. The inconsiderable impact of attitudes towards the epidemic can be attributed to an acknowledgement of and trust in national containment and control policies and measures, as well as to the existence of similar perceptions, such as the SARS pneumonia in 2003.

## Figures and Tables

**Table 1:** Analysis of the demographic characteristics of the survey sample.

		Proportion/%			Proportion/%
Gender	Male	45.4	Exercise time after the outbreak (adult group)	>300 min of moderate aerobic exercise/>150 min of vigorous exercise	11.2
	Female	54.6		150–300 min of moderate aerobic exercise/75–150 min of vigorous exercise	50.5
Age	Under the age of 18	9.1		<150 min of moderate aerobic exercise/<75 min of vigorous exercise	38.3
	18–25	2.2	During home confinement, it is better to be physically active than to remain sedentary	Strongly agree	31.8
	26–30	7.6		Agree	50
	31–40	56.6		Commonly	12.2
	41–50	21.8		Disagree	1.7
	51–65	2.3		Strongly disagree	4.3
	Age more than 65 years	0.1	Exercise habits before and after the outbreak	Had before, stopped after the outbreak	7.9
Education	Primary school	4.1		Both before and after the outbreak	68.2
	Junior high school	18.2		Did not have before and started after the outbreak	16.8
	Senior high school	30.1		Null before and after the outbreak	7
	College and above degree	45.6	Attention paid to fitness-related indices	Reduce a lot of attention	4.4
	Master’s degree or above	1.7		Reduce some of the attention	5.8
Exercise time before the outbreak (adult group)	>300 min of moderate aerobic exercise/>150 min of vigorous exercise	9.8		Uniformity	20.3
	150–300 min of moderate aerobic exercise/75–150 min of vigorous exercise	40.6		Add some attention	22.8
	<150 min of moderate aerobic exercise/<75 min of vigorous exercise	49.6		Add a lot of attention	46.7

**Table 2:** Descriptive statistics and correlation coefficients of key variables.

Variable		M	SD
V1	PA intentions	3.944	0.708
V2	Attitudes: apps	3.980	0.728
V3	Attitudes: epidemic	3.545	0.853
V4	Attitudes: exercise	3.953	0.654

Note. M, mean; SD, standard deviation; male = 1.

**Table 3:** Multiple linear regression of intention to use exercise behaviours (or PA) to resist COVID-19.

Model I			
Step	Variable Entered	β	β
1	Gender (male = 1)	0.592 ***	0.149 ***
	Age	0.007	0.017
	Education	0.036	0.017
2	Attitudes towards exercise		0.822 ***
	N	1223	1223
	R/adjusted R^2^	0.35/0.35	0.83/0.83
	ΔR^2^	0.35	0.48
	ΔF	221.57 ***	3467.31 ***
	Model F	221.57 ***	1505.53 ***
**Model II**			
**Step**	**Variable Entered**	**β**	**β**
1	Gender (male = 1)	0.592 ***	0.378 ***
	Age	0.007	0.021
	Education	0.036	0.004
2	Attitudes towards apps		0.526 ***
	N	1223	1223
	R/adjusted R^2^	0.35/0.35	0.58/0.58
	ΔR^2^	0.35	0.23
	ΔF	221.57 ***	671.95 ***
	Model F	221.57 ***	425.63 ***
**Model III**			
**Step**	**Variable Entered**	**β**	**β**
1	Gender (male = 1)	0.592 ***	0.571 ***
	Age	0.007	0.055 *
	Education	0.036	0.003
2	Attitudes towards the epidemic		0.196 ***
	N	1223	1223
	R/adjusted R^2^	0.35/0.35	0.39/0.39
	ΔR^2^	0.35	0.04
	ΔF	221.57 ***	75.08 ***
	Model F	221.57 ***	195.04 ***

Note: * *p* < 0.05, *** *p* < 0.001.

**Table 4:** Multiple linear regression of intention to use exercise behaviours (or PA) to resist COVID-19.

Model IV				
Step	Variable Entered	β	β	β
1	Gender (male = 1)	0.592 ***	0.149 ***	0.149 ***
	Age	0.007	0.017	0.015
	Education	0.036	0.017	0.018
2	Attitudes: exercise		0.822 ***	0.778 ***
3	Attitudes: apps			0.056 ***
	Attitudes: epidemic			0.016
	N	1223	1223	1223
	R/adjusted R^2^	0.35/0.35	0.83/0.83	0.83/0.83
	ΔR^2^	0.35	0.48	0.002
	ΔF	221.57 ***	3467.31 ***	7.19 ***
	Model F	221.57 ***	1505.53 ***	1016.28 ***

Note: *** *p* < 0.001.

## Data Availability

The data presented in this study are openly available by contacting the corresponding Author.
